# Structural vaccinology-based design of multi-epitopes vaccine against *Streptococcus gordonii* and validation using molecular modeling and immune simulation approaches

**DOI:** 10.1016/j.heliyon.2023.e16148

**Published:** 2023-05-11

**Authors:** Syed Nouman Nasir, Ayesha Iftikhar, Farukh Zubair, Abdulrahman Alshammari, Metab Alharbi, Abdullah F. Alasmari, Abbas Khan, Muhammad Waseem, Syed Shujait Ali, Liaqat Ali, Yasir Waheed, Dong-Qing Wei

**Affiliations:** a National Center for Bioinformatics, Quaid-i-Azam University, Islamabad, Punjab, Pakistan; b Government Khwaja Muhammad Safdar Medical College, Sialkot, Punjab, Pakistan; c Rashid Latif Medical College, Lahore, Punjab, Pakistan; d Department of Pharmacology and Toxicology, College of Pharmacy, King Saud University, Post Box 2455, Riyadh, 11451, Saudi Arabia; e Department of Bioinformatics and Biological Statistics, School of Life Sciences and Biotechnology, Shanghai Jiao Tong University, Shanghai, 200240, PR China; f Zhongjing Research and Industrialization Institute of Chinese Medicine, Zhongguancun Scientific Park, Meixi, Nayang, Henan, 473006, PR China; g Faculty of Rehabilitation and Allied Health Science, Riphah International University, Islamabad, Pakistan; h Fisch College of Pharmacy, The University of Texas at Tyler, Tyler, TX, USA; i Office of Research, Innovation, and Commercialization (ORIC), Shaheed Zulfiqar Ali Bhutto Medical University (SZABMU), Islamabad, 44000, Pakistan; j Gilbert and Rose-Marie Chagoury School of Medicine, Lebanese American University, Byblos, 1401, Lebanon

**Keywords:** *Streptococcus gordonii*, Immunoinformatics, Multi-epitopes vaccine, Molecular docking, Immune simulation

## Abstract

*Streptococcus gordonii* is an oral bacterium colonizing the dental cavity and leading to plaque formation. This pervasive colonizer is also the etiologic agent of bacterial endocarditis and has a major role in infective endocarditis. The bacteria reach the heart through oral bleeding, leading to inflammation of cardiovascular valves. Over the past 50 years, it has shown a significant pathogenic role in immunocompromised and neutropenic patients. Since antibiotic resistance has created prophylaxis failure towards infective endocarditis, a potent therapeutic candidate is needed. Therefore, multi-epitopes vaccine offers advantages over the other approaches. Thus, herein, numerous molecular-omics tools were exploited to mine immunogenic peptides, i.e., T-cell and B-cell epitopes, and construct a vaccine sequence. Our findings revealed a total of 24 epitopes, including CTL, HTL, and B-cell are responsible for imparting immune responses, which were combined with the help of different linkers, and MEVC was constructed. Multifactorial validation of the candidate vaccine was performed to minimize the risk factors. The final sequence was docked with TLR2 to validate its conformation compatibility with receptor and long-term interactions stability. Our analysis revealed that the vaccine construct is immunogenic and non-allergenic. The construct also established various contacts with the immune receptor. Finally, the vaccine sequence was reverse-translated, optimized for codon usage, and analyzed for expression in the *Escherichia coli* K12 strain. Maximum expression was noted with a CAI score of 0.95. *In silico* immune simulation revealed that the antigen was neutralized on the 3rd day after injection. In conclusion, the current study warrants validation of the vaccine construct both in *in vitro* and *in vivo* models for accurate therapeutic intervention.

## 1 Introduction

Viridian streptococci, comprising *Streptococcus Gordonii,* is a gram-positive oral bacterium that colonizes the dental cavity leading to plaque formation [1]. These oral bacteria cause several oral infections and can result in tooth loss. It was first reported in 1946 in a patient with infective endocarditis, however, announced in 1989 as a type strain with approximately 2.1 million base pair nucleotides [2]. Among the initial settlers of the periodontal environment*, Streptococcus gordonii* holds a dominant place. These pervasive initial colonizers represent most of the cultivable bacteria found in dental plaque and are the recurring etiologic agents of bacterial endocarditis, which is the inflammation and damage of cardiovascular valves, a severe infection resulting in a high fatality rate despite the deep clinical and surgical comprehensions [[3], [4], [5], [6]]. It reaches the heart through the bloodstream using oral bleeding [7]. Previously in-vivo reported Hsa/GspB, a serine-rich surface protein that binds to platelet cells through fibrinogen and fibronectin bridging molecules. Thus, is the most important protein in primarily stimulating the infection cascade [8]. Fibrillar proteins CshA and CshB are involved in binding to fibronectin molecules of humans and proceed the infection, followed by surface protein A, responsible for secondary adhesion (SspA). Over the last decades, it has shown a significant pathogenic role in immunocompromised and neutropenic patients [9,10]. The alarming aspect of *Streptococcus gordonii* causing Infective Endocarditis (IE) is that one-half of the patients are infected with no prior heart-associated disease [11]. It is now affecting 3–10 individuals in 10 thousand and recent consensus research are indicating the rise in number [12,13]. Also, about one in four patients are suffered from neurological complications [14].

*Streptococcus gordonii* is one of the causative agents of IE has different morbidity and mortality rates in low, middle, and high-income countries. Several prior heart and rheumatic diseases contribute to its epidemiology [15]. The highest mortality rate is found in South Latin America, and Eastern Europe, followed by East Asia. About a 40% mortality rate is recorded in 5 years, with 22% in Hospital cases [16,17]. In rural upstate New York from 2011 to 2016, in a total population, 4.4 cases were recorded in 100 thousand personnel with a total number of 45 confirmed patients and 9 possible [18]. Another epidemiologic research was carried out in an urban area with 428,000 personnel in a 5 years interval; the ratio of the cases came up to 6.2 in 0.1 million individuals [19]. A 17-year health record in Italy revealed a 24% of mortality rate in-hospital patients, mostly had prior heart-associated and rheumatic diseases [20]. The treatment for IE varies for different aged individuals; however, certain general antibiotic therapies are prescribed, including a combination of aminoglycoside and vancomycin [3]. The recommendation of surgical therapeutics for IE patients is multifaceted and therefore, surgeries are performed in 40%–45% of patients [3]. Antibiotic resistance is creating major failures toward infective endocarditis prophylaxis [21]. *Streptococcus gordonii* has been used as a live oral vaccine vector for the control of the *Schistosoma japonicum* worm. *Streptococcus gordonii* expressing M6-Sj-F1 fusion protein protected mice from infection caused by *Schistosoma japonicum* [22]. Therefore, the best alternative treatment is a vaccine which is reported to save millions of lives yearly [23]. Numerous molecular-omics tools were exploited to mine immunogenic peptides, i.e., T-cell and B-cell epitopes, and construct a vaccine sequence. Our study revealed a total of 24 epitopes including CTL, HTL, and B-cell are responsible for imparting immune responses. Multifactorial validation of the candidate vaccine was performed to minimize the risk factors. The final sequence was docked with TLR2 to validate its conformation compatibility with receptor and long-term interactions stability. Consequently, the vaccine sequence was reverse translated, optimized for codon usage, and analyzed for expression in the *Escherichia coli* K12 strain. Maximum expression was noted with a CAI score of 0.95. Furthermore, the current study warrants validation in *in vivo* models for accurate therapeutic intervention.

## 2 Materials and methods

### 2.1 Retrieval and selection of proteins

**Fig. 1:**
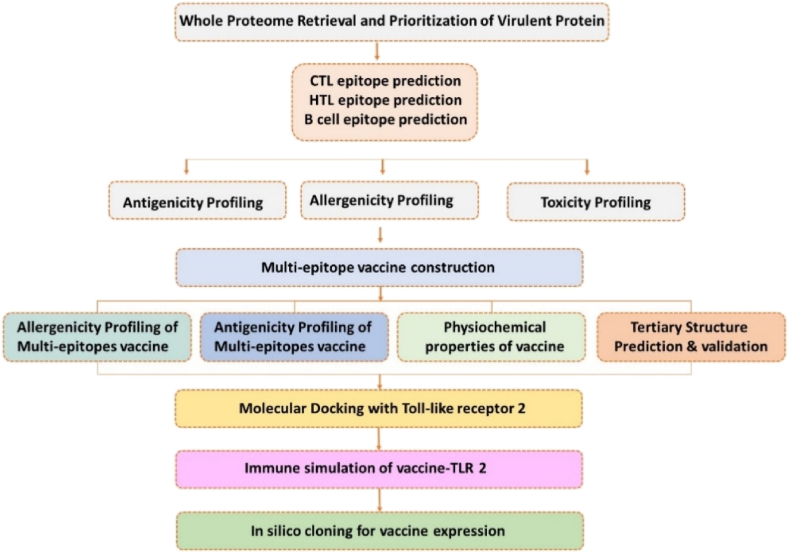
The methodological workflow for designing a multi-epitopes subunit vaccine using immuno-informatics approaches.

The proteome of *S. gordonii* (strain challis) UniProt ID (UP000001131) and gene bank accession ID = PRJNA66 was retrieved from an online data server, Universal Protein Resource (https://www.uniprot.org/) to prioritize proteins for vaccine designing [24]. Literature-based prioritization of the best proteins involved in *S. gordonii-*associated infection were retrieved. The priority was given to a serine-rich protein GspB, having UniProt ID (Q939N5) due to major involvement in the pathogenesis of infective endocarditis [8]. The next priority was given to fibrillar proteins CshA and CshB having UniProt ID (A8AWJ3) and (A8AXC5), respectively, due to involvement in binding to fibronectin molecule of humans [25]. The final selected protein was surface protein A SspA having UniProt ID (A8AUS0), which is involved in the secondary adhesion [25]. All the 4 proteins were collected from the proteome of *S. gordonii* [26]. The overall workflow of the work is given in Fig. 1.

### 2.2 Data processing

#### 2.2.1 Prediction of MHC-I epitopes

The function of cytotoxic T lymphocyte (CTL) is to diminish the antigen upon recognition with the help of a helper T lymphocyte. For all the four selected proteins, CTL epitopes against MHC-I were predicted by utilizing an online web server at (https://services.healthtech.dtu.dk/services/NetCTL-1.2/) with a threshold of 0.75 [26]. A high combined score shows a high binding affinity. The predicted result is based on the binding affinity of CTL epitopes to MHC- I, proteasomal C terminus cleavage score, and transport efficacy of transporter associated with antigen processing. The score of TAP was enumerated by a weight matrix, while MHC-I binding and proteasomal C terminus cleavage were by an artificial neural network. Final epitopes were selected after passing them through parameters like antigenicity, allergenicity, toxicity, and immunogenicity.

#### 2.2.2 Prediction of MHC-II epitopes

The helper T lymphocytes induce killer T cells and stimulate humoral immunity to act against the antigen. Therefore, it holds a critical role in prophylactic and immunotherapeutic vaccines. An online web server, Immune Epitopes Database (IEDB) (http://www.iedb.org/) [26,27] was exploited for the prediction of Helper T Lymphocyte HTL epitopes with reference to the Human leukocytes named as HLA-DRB1*03; 01, HLA-DRB3*01; 01, HLA-DRB1*15; 0, HLA-DRB1*07; 01, HLA-DRB4*01; 01, HLA-DRB3*02; 02 and HLADRB5*01; 01 for the prioritized proteins. The server allocates the IC50 value to the epitopes, which has an inverse relation with the binding affinity towards MHC-II. IC50 score of less than 50 nM constitutes high binding affinity, a score less than 500 nM is determined as intermediate binding affinity and less than 5000 nM as low binding affinity [23]. Moreover, the HTL epitopes were selected based on a low percentile rank that indicates their high binding affinity.

#### 2.2.3 Prediction of B-cell epitopes

B cell epitopes are recognized by receptors at B lymphocyte and thus bind to it. We exploited ABCpred, an online server (http://www.imtech.res.in/raghava/abcpred/) for the prediction of linear B cell epitopes, having 75% accuracy (0.75 specificities and 0.49 sensitivity) at a 3.7 default threshold [28]. Further evaluation for antigenicity, toxicity, and allergenicity was ascertained by VaxiJen v2.0, ToxinPred, and AlgPred servers, respectively [[29], [30], [31]].

#### 2.2.4 Interferon‐γ epitope prediction

Interferon-gamma proteins are involved in the activation of macrophages, mediation of antiviral and antibacterial immunity, enhancement of antigen presentation, and activation of the innate immune system; thus are of great importance in vaccine designing [23]. An online web server (http://crdd.osdd.net/raghava/ifnepitope/) was exploited for the prediction of epitopes that induce interferon-gamma cells [32]. Non-allergenic B cell epitopes were prioritized with high scores, which would contribute to the non-allergenic nature of the final vaccine sequence. This server utilizes motifs and a hybrid algorithm of support vector machine SVM for the prophecy of interferon-producing properties of the MHC-II epitopes. It also assigns an SVM score for each epitope.

### 2.3 Multi-epitopes vaccine construct (MEVC) and validation

#### 2.3.1 Vaccine construction

The vaccine sequence was constructed from immunogenic peptides. HTL and CTL epitopes were carefully assessed based on having the best values in non-allergenicity, antigenicity, and non-toxicity scores. Further, CTL epitopes with high immunogenicity scores and HTL with the best score in inducing interferon-gamma cells were selected. AAY linkers for the fusion of CTL epitopes and GPGPG for HTL were used. Among multifunctional features of linkers include hindrance of self-folding of epitopes, enhancement of immunity, and presentation to MHC-I and MHC- II [[33], [34], [35], [36]]. B cell epitopes were fused right after HTL epitopes with KK linkers. The Combined ratio for epitopes was 2:3:1 (HTL, CTL, and B cell), respectively, except for CshB protein with 3:2:1. Moreover, to intensify the immunogenicity of the vaccine, Pam3CSK4 (Pam3CysSerLys4) is a synthetic triacylated lipopeptide (LP) chain C (PDB ID; 2Z7X), TLR2 agonist, was used [37]. The linker EAAAK was used to fuse the adjuvant at N-terminus [38].

#### 2.3.2 Prediction of allergenicity

Allergenicity prediction of the vaccine construct with an accuracy up to 85% was attained through an online server, the Algpred (http://www.imtech.res.in/raghava/algpred/) [39]. The server exploits a hybrid algorithm to ensure the non-allergenic nature of a vaccine sequence at a threshold of −0.4. About six different methods are utilized by the server, namely the MEME/MAST motif, mapping of the IgE antigenic peptides, PID, and the blast search on the ARPs (allergen representative peptides). Two support vector machine approaches are utilized based on the composition of amino acids and the composition of dipeptides.

#### 2.3.3 Prediction of antigenicity

To predict the antigenicity of the vaccine construct, we exploited the VaxiJen server (http://www.ddg-pharmfac.net/vaxijen/VaxiJen/VaxiJen.html) [29]. The server merely predicts the score based on the given amino acids’ sequence physio-chemical properties as an alternative using a sequence alignment algorithm. The VaxiJen server enumerates the antigenicity value with accuracy up to 70–90%.

#### 2.3.4 Physiochemical properties

An online web tool, ProtParam (http://web.expasy.org/protparam/) [40] was employed for the enumeration of different physiochemical properties of vaccine sequence, that are, the constitution of amino acid, instability index, theoretical PI, half-life *in vivo* and in vitro, aliphatic index, and grand average of hydropathy (GRAVY). For the prediction of MEVC solubility, Protein-sol (http://protein-sol.manchester.ac.uk/) was used to determine the solubility of the designed construct.

#### 2.3.5 Secondary and tertiary structure prediction

To determine the protein folding, online servers such as SOPMA (https://npsa-prabi.ibcp.fr/cgi-bin/npsa_automat.pl?page=/NPSA/npsa_sopma.html) and PSIPREDV3.3 (http://bioinf.cs.ucl.ac.uk/psipred/) were employed to predict the secondary structure of the vaccine as it is one of the significant steps of the procedure [41]. Robetta (http://robetta.bakerlab.org) an online web server, was made use of to serve the generation of the tertiary structure of vaccine [42]. It examines the constructed protein sequences into supposed domains. In the case of recognition of template structure for the query protein sequence, utilize PSI-BLAST, BLAST, FFAS03, or 3D-Jury, hence, leads to the generation of a structure employing comparative modeling technique. On the contrary, the de-novo Rosetta fragment insertion technique is exploited. Moreover, to refine the tertiary structure of the vaccine, we utilized an online server Galaxy Web (https://galaxy.seoklab.org/) [43].

#### 2.3.6 Validation of tertiary structure of vaccine

Since validation is one of the crucial steps, we exploited different online servers. It ensures the authenticity of the 3D model of the protein. ProSA-web server (https://prosa.services.came.sbg.ac.at/prosa.php) was exploited to compute the Z-score of the respective 3D structure [44]. It indicates the chances of accuracy and inaccuracy of protein to appear in the required range established for natural protein. ProSA-Web server also validates the troublesome part and elucidates it in a plot score by means of a 3D model viewer. Another online web server ERRAT (http://services.mbi.ucla.edu/ERRAT/), was utilized to calculate the non-bondage occurring in 3D structure [45]. Ramachandran plot of the vaccine construct was evaluated using the PROCHECK server. (https://saves.mbi.ucla.edu/).

#### 2.3.7 Docking the vaccine's tertiary structure with human TLR-2

We exploited one of the widely used servers ClusPro (https://cluspro.bu.edu/login.php), for docking the receptor and ligand. The final vaccines 3D model was docked with Human Toll-like receptor 2 as it has been reported to possess adjuvant properties and is crucial in imparting cellular and humoral immune mechanisms in diﬀerent animal species [46,47]. It predicted 10 docking results, each having parameters based on highly dense clusters with the lowest energy. This further elucidates the exploitation of numerous options such as constructing secondary imitation files, selection of multiple energy constraints, and output analysis. ClusPro generates the outputs in about 4 h. Moreover, we exploited the PDBsum server to generate the graphical view of vaccine-TLR2 interacting residues.

#### 2.3.8 Immune simulations

We used the C-ImmSim server (https://kraken.iac.rm.cnr.it/C-IMMSIM/index.php?page=1) to predict the immune response of a human against antigens through an agent-based modeling method [48]. The PSSM approach of the server represents the immune response. Right after injection of the vaccine construct determines the production of antibodies, interferons, and cytokines. Furthermore, it predicts the responses of Th1 and Th2. A plot of the Simpson Index or D (a measure of diversity) was used with default parameters.

#### 2.3.9 Optimization of codons and in-silico cloning

An online web tool Jcat was utilized to serve the reverse translation of peptide sequence and its optimization to ensure certainty of expression in a selected vector [49]. We selected an option to avoid cleavage sites for restriction enzymes to overcome the formation of restriction sites in the optimized sequence. Another option to avoid Rho-independent termination of transcription was opted to escape premature transcription termination. The server estimated the number of GC residues and codon adaption index (CAI) for the moderated reversed sequence to approximate the level of expression in the *E. coli* system. *Xho*I at N-terminus while EcoR1 at C-terminus were manually inserted into the vaccine sequence at restriction sites. Snapgene Software was utilized to insert the final sequence into the pET-28+ plasmid to serve its cloning.

## 3 Results

### 3.1 Retrieval of the proteome and selecting proteins for B and T-cell epitopes prediction

The whole proteome was downloaded from UniProt, and prioritization of crucial proteins was performed through a literature search. The UniProtKB option was used for sequences retrieval. Based on previously reported in-vivo, four pathogenic proteins were selected, which are GspB, serine-rich surface glycoprotein having UniProt ID (Q939N5), CshA, UniProt ID (A8AWJ3), CshB UniProt ID (A8AXC5), and the surface protein A SspA having UniProt ID (A8AUS0), for vaccine designing. The peptide sequences were subjected to B and T cell epitopes prediction to design a multi-epitopes subunit vaccine.

### 3.2 Antigenicity prediction

The VaxiJen server enumerated the antigenicity scores for GspB, SspA, CshA, and CshB protein as 1.39, 0.66, 0.70, and 0.72, respectively at a threshold of 0.4 as shown in **(**Table 1**).** These scores were above 0.4, which is the minimum threshold for antigenicity on the VaxiJen server. The antigenic nature of the proteins ensured that their use in subunit vaccine design could induce substantial humoral immune responses, which in turn could stimulate cellular immunity to destroy the antigen.

**Table 1:** Candidate proteins for vaccine designing retrieved from the UniProt server.

S·NO	Protein name	UniProt ID	Antigenicity Score
1	Platelet binding protein (GspB)	Q939N5	1.39
2	Surface protein A (SspA)	A8AUS0	0.66
3	Surface-associated protein (CshA)	A8AWJ3	0.70
4	Surface-associated protein (CshB)	A8AXC5	0.72

### 3.3 Prediction of MHC-I epitopes

NETCTL1.2 server forecasted 23, 46, 47, and, 52 CTL epitopes (9-mer) for GspB, SspA, CshA, and CshB, respectively. A total of 9 MHC-I binding epitopes were selected for the vaccine construct. Two epitopes each from GspB, SspA, and, CshA while 3 from CshB protein with antigenic, non-toxic, and immunogenic nature were finalized as shown in **(**Table 2**).** The epitope's interaction with MHC alleles will allow recognition by the T cell receptors, followed by the initiation of immune responses. The antigenicity for each epitope was predicted to confirm the immunogenic effects of these epitopes and presented in Table 2. The server predicted all epitopes as antigenic and suitable for usage to construct the MEVC.

**Table 2:** Selected CTL epitopes for vaccine construct predicted by NetCTL 1.2.

Uniport ID	Residues no	Peptide sequence	MHC Binding Score	C-terminal cleavage affinity	TAP	Prediction score	MHC Binder	Antigencity scores
**Q939N5**	179	ASASESLSV	0.15	0.91	0.57	0.81	Yes	1.18
	81	AVVTSSSVY	0.17	0.97	3.25	1.04	Yes	0.41
**A8AUS0**	100	VTDAKAAGV	0.26	0.76	0.33	1.25	Yes	1.18
	980	VVPTVHFHY	0.22	0.97	2.96	1.24	Yes	1.17
**A8AWJ3**	1803	VTATYTPTV	0.18	0.89	0.20	0.94	Yes	1.01
	2486	LAAIASLTF	0.13	0.79	2.65	0.82	Yes	0.53
**A8AXC5**	2092	RLDIYGNVV	0.16	0.95	0.50	0.85	Yes	0.67
	1298	VTATYTPSV	0.14	0.85	0.20	0.76	Yes	1.02
	367	TTNGTGWEY	0.72	0.93	2.68	3.34	Yes	1.52

### 3.4 Prediction of MHC-II epitopes

IEDB MHC-II server predicted MHC-II binding epitopes against a reference set of 7 human HLAs for the prioritized proteins. Eleven non-overlapping MHC-II binding epitopes were selected having the lowest percentile rank, inducing interferon-gamma, non-toxic and non-allergenic properties. The epitopes finalized from GspB, SspA, and CshA were 3 in number, while 2 epitopes were selected from CshB as given in **(**Table 3**).**

**Table 3:** Selected HTL epitopes for vaccine construct predicted by IEDB MHC-II.

UniProt ID	S.No	HLAs	start	end	Epitope Chain	Percentile Rank
**Q939N5**	1	HLA-DRB5*01:01	510	524	TSVNGQFKLIIRFRI	1.40
	1	HLA-DRB3*02:02	84	98	LTPRQVKSNLGALVS	1.00
	1	HLA-DRB3*02:02	276	290	RLLTWTINLTPRQVK	0.23
**A8AUS0**	14	HLA-DRB5*01:01	24	38	KRPNIWYSLNGKIRA	1.40
	25	HLA-DRB3*01:01	2	16	SLDSPFQAETYLQMR	15.00
	13	HLA-DRB3*02:02	46	60	WYGAGAISMSGPTNH	28.00
**A8AWJ3**	1	HLA-DRB4*01:01	8	22	PHLRKFSIRKLNVGV	1.50
	12	HLA-DRB3*02:02	40	54	RSDFVTNASMSNQAP	0.01
	13	HLA-DRB1*07:01	25	39	ASPETVSPSRATDQP	51.00
**A8AXC5**	3	HLA-DRB1*15:01	15	29	PEQANARYLKRVGAA	38.00
	3	HLA-DRB5*01:01	41	55	ATTPASKEEAKKSEA	60.00

### 3.5 Prediction of B-cell epitopes

Antibody-mediated immunity aids in neutralizing the circulating antigen from the pathogen by binding to the B-cell receptor, allowing the B cells to mature in plasma cells for producing specific immunoglobulins. ABCpred server predicted epitopes of B-cell for each candidate protein, among which a single epitope with non-allergenic, antigenic nature and the highest score allotted by the server was selected for vaccine construct that further increased the antigenicity and non-allergenicity of vaccine sequence. From the total predicted B-cell epitopes of GspB, (SASQSMHDRISKGQLP) starting at position 3024 was finalized. Likewise, (GDPAKTPVTPDASRPA) from CshA (QVSDTDGKAHRARYQP) from CshB, and (KKEVEAHQAETDKINA) from SspA starting at position 829, 885, and 153 respectively. The final epitopes are given in **(**Table 4**).**

**Table 4:** Linear B cell epitopes predicted by ABCpred server.

Protein	Sequence	Starting position	Score
GspB	SASQSMHDRISKGQLP	3024	0.89
CshA	GDPAKTPVTPDASRPA	829	0.96
CshB	QVSDTDGKAHRARYQP	885	0.94
SspA	KKEVEAHQAETDKINA	153	0.85

### 3.6 Prediction of IFN‐γ epitopes

IFN-epitope, an online web server, predicted the MHC-II binding epitopes. 3 out of 11 HTL epitopes were found to induce interferon-gamma protein. Among 3 HTL epitopes of Platelet binding protein (GspB) only one epitope (RLLTWTINLTPRQVK) was classified as IFN positive. Similarly (KRPNIWYSLNGKIRA) and (PHLRKFSIRKLNVGV) epitopes for surface protein A (SspA) and surface-associated protein A (CshA) respectively reported being IFN positive. However, all the epitopes of surface-associated protein B (CshB) resulted in IFN negative. The epitopes with scores are given in **(**Table 5**).**

**Table 5:** Interferon-gamma epitopes prediction by IFN-epitope server.

UniProt ID	S. No	Peptide sequences	Method	Result	Score
**Q939N5**	1	TSVNGQFKLIIRFRI	SVM	NEGATIVE	−0.43
	2	LTPRQVKSNLGALVS	SVM	NEGATIVE	−0.33
	3	RLLTWTINLTPRQVK	SVM	**POSITIVE**	0.14
**A8AUS0**	4	KRPNIWYSLNGKIRA	SVM	**POSITIVE**	0.73
	5	SLDSPFQAETYLQMR	SVM	NEGATIVE	−0.13
	6	WYGAGAISMSGPTNH	SVM	NEGATIVE	−0.35
**A8AWJ3**	7	PHLRKFSIRKLNVGV	SVM	**POSITIVE**	0.07
	8	RSDFVTNASMSNQAP	SVM	NEGATIVE	−0.41
	9	ASPETVSPSRATDQP	SVM	NEGATIVE	−0.21
**A8AXC5**	10	PEQANARYLKRVGAA	SVM	NEGATIVE	−0.04
	11	ATTPASKEEAKKSEA	SVM	NEGATIVE	−0.60

### 3.7 Vaccine construction

**Fig. 2:**
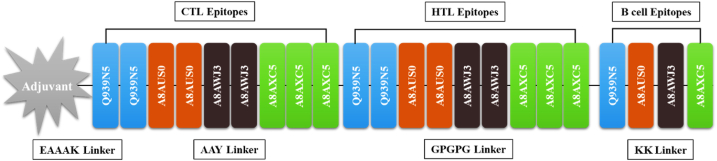
Figure representing epitopes and adjuvant arrangement in vaccine sequence. The red color represents EAAAK linker, the blue color represents Platelet binding protein (GspB), the brown color represents Surface protein A (SspA), the black color represents Surface-associated protein (CshA) and the green color represents the Surface-associated protein (CshB). (For interpretation of the references to color in this figure legend, the reader is referred to the Web version of this article.)

We constructed our vaccine structure from 9 CTL, 11 HTL, and, 4 B-cell epitopes based on their high affinity towards MHC-I and MHC-II respectively. A High COMB score indicates a higher affinity of CTL towards MHC-I, while a low percentile rank indicates a higher affinity of HTL towards MHC-II. The selected CTL, HTL, and, B-cell epitopes were fastened together by AAY, GPGPG, and KK linker, respectively, to refrain from mislinkage and amplify flexibility. Also, the linkers keep the epitopes and adjuvant separated from each other so that they can be efficiently recognized by the host immune system for processing and generating specific responses. The mammalian beta-defensin was fused to the N-terminus of the vaccine sequence using the EAAAK linker to function as an adjuvant to boost immune response. The use of adjuvant allows to multiply the antigenic potential of the multi-epitopes vaccine contrast as individual epitope has low antigenicity. The order of arrangement of Adjuvant, CTL epitopes, HTL epitopes, B-cell epitopes, and, Linkers in the final vaccine sequence that consists of 407 amino acids are depicted in (Fig. 2). The combined epitopes containing both B-cell and T-cell epitopes make the construct act as both antigenic and immunogenic.

### 3.8 Prediction of allergenicity and antigenicity

An antigenic score of 1.02 was obtained from the VaxiJen server for the constructed vaccine at a threshold of 0.4, ensuring its capacity to provoke the immunity of the host. AlgPred server ensured the non-allergenic nature by calculating a value of −0.87 at a threshold of −0.4. This further confirmed the safe nature.

### 3.9 Prediction of physicochemical properties

Protparam web server generated physio-chemical properties of a vaccine construct as shown in **(**Table 6**).** The theoretical protrusion index (PI) with a score of 10.03 and molecular weight of 42.15 KDa confirmed the basic nature of the vaccine. An instability index of less than 40 indicates the constructed sequence is stable. Our construct was found to score 26.31. The half-life (*in vivo*) of vaccine constructs in *E. coli* and *Yeast* is > 10 and > 20 h, respectively which explains this protein sequence is not purified for vaccine production. Meanwhile, it is 30 h in mammalian reticulocytes (in vitro). The Grand Average Hydropathy (GRAVY) score of the vaccine was −0.49, indicating its hydrophilic nature. Furthermore, the value of the aliphatic index of 61.74 validated the thermostability of the vaccine construct. In consequence, our vaccine construct evinces properties best for triggering an immune response and downstream process. The solubility analysis revealed 0.746 as the scaled solubility for our vaccine construct which is larger than the threshold of 0.45 and thus demonstrates that the vaccine construct is soluble compared to the average solubility of *E. coli* proteins.

**Table 6:** Physiochemical properties of final vaccine construct.

S·NO	Property	Score	Outcome
**1**	Allergenicity	−0.87 (threshold −0.4)	Non-allergenic
**2**	Antigenicity	1.02 (threshold 0.4)	Antigenic
**3**	Molecular weight	42.15 KDa	–
**4**	Theoretical PI	10.03	Basic
**5**	Half life	>10 h in *E. coli*	–
**6**	Instability index	26.31 (stable)	Stable
**7**	Aliphatic index	61.74	–
**8**	GRAVY	−0.49	Hydrophilic
**9**	Solubility	0.746	Soluble

### 3.10 Prediction and validation of tertiary structure

**Fig. 3:**
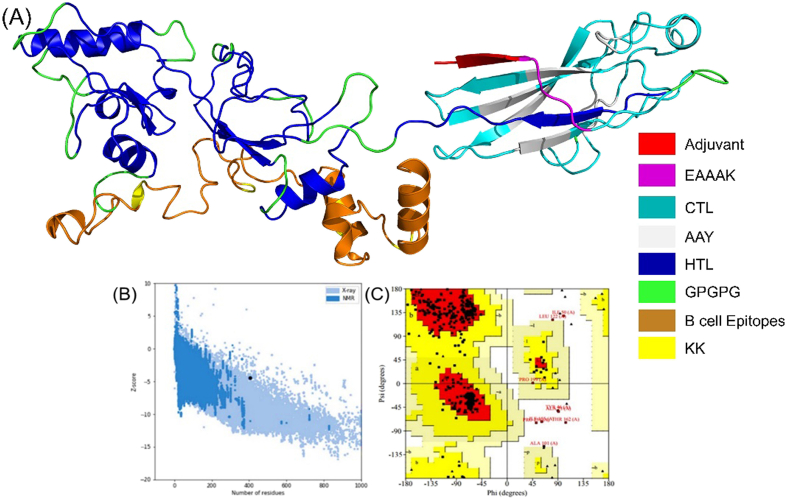
**(A)** Vaccine's final structure depicting Helix, loop, and beta-sheets. Validation of finalized vaccine 3D model. **(B)** Validation by ProSA-web of 3D structure (Z-score: 4.48) **(C)** Ramachandran assessment illustrating the residues (Black squares and triangles): 87.9% in most favored (Red), 9.9% in additionally allowed (Bright Yellow), 0. % in generously allowed (Light Yellow) and 2.2% in disallowed regions (White). (For interpretation of the references to color in this figure legend, the reader is referred to the Web version of this article.)

We exploited the Robetta server that generated five models for the given vaccine sequence. Model 3 was selected for further analysis after a detailed assessment such as C score, ERRAT, ProSA-web, and PROCHECK scores, as shown in (Fig. 3A). Galaxy Web server further refined the structure. To validate the structure, we exploited ERRAT, ProSA-web, and PROCHECK servers to spot and settle viable errors in the vaccines’ 3D structure. ERAAT server resulted in a quality score of 80.5 for the given 3D structure. ProSA-Web server resulted in the overall model quality of the 3D structure with a Z-score of −4.48, which is within the standard score for protein. The Ramachandran plot, assessed via the PROCHECK server, led to resulting 87.9% residues in the most favored region, 9.9% residues in the additional allowed region, 2.2% in the disallowed region, and, 0% generously allowed region as shown in (Fig. 3B and C).

### 3.11 Predictions of secondary structure

**Fig. 4:**
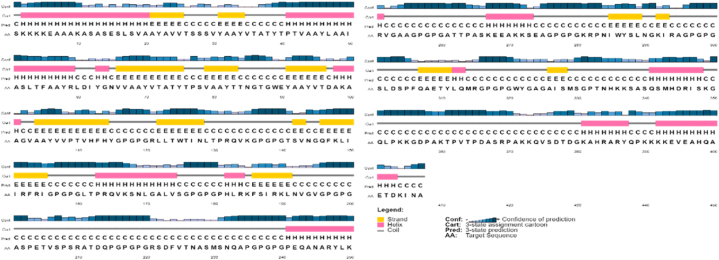
Figure illustrating secondary structure predicted by PSIPREDV3.3. SOPMA. It predicted 18.18% extended strands, 27.76% alpha-helix, 4.18% beta turns, and 49.88% random coils.

SOPMA and PSIPRED servers anticipated the secondary structure of the final vaccine sequence. The result showed 18.18% extended strands, 27.76% alpha-helix, 4.18% beta turns, and 49.88% random coils as shown in (Fig. 4).

### 3.12 Docking the vaccine's tertiary structure with human TLR-2

**Fig. 5:**
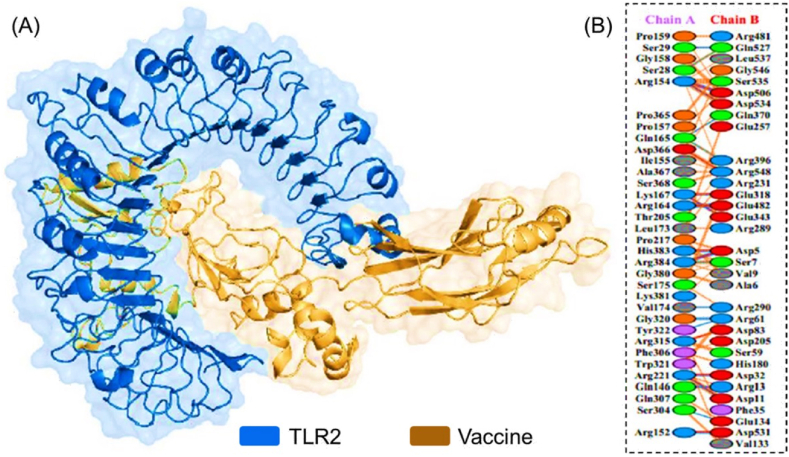
**(A)** Vaccine-TLR-2 complex. The Toll-Like Receptor-2 (receptor) is shown in Orange, while the magenta color shows the vaccine. **(B)** PDBsum file represents blue, red, and orange-colored lines as H-bonds, Salt bridges, and non-bonded contacts, respectively between (Chain A: TLR2) and (Chain B: Vaccine). (For interpretation of the references to color in this figure legend, the reader is referred to the Web version of this article.)

After submitting the constructed vaccine and human TLR-2 PDB structure the ClusPro docking server resulted in 10 complexes of Human TLR2 and vaccine interaction based on its cluster size. PyMOL software was employed to inspect and evaluate the docking complexes. Consequently, complex 10 was finalized for further analysis. The server assigned a weighted score of −724.8 center and −937.3 lowest. PDBsum server delivered the graphical representation of the residues interaction between TLR2-Vaccine, and a graphical image of hydrogen bonds and salt bridges. In total, 24 hydrogen bonds were found between TLR2-Vaccine along with 12 salt bridges followed by 220 non-bonded contacts. The graphical image (3D) of the interacted complex is shown in Fig. 5A, whereas Fig. 5B illustrates the hydrogen bonds and salt bridges between Human TLR2 and the vaccine.

### 3.13 Immune simulation

**Fig. 6:**
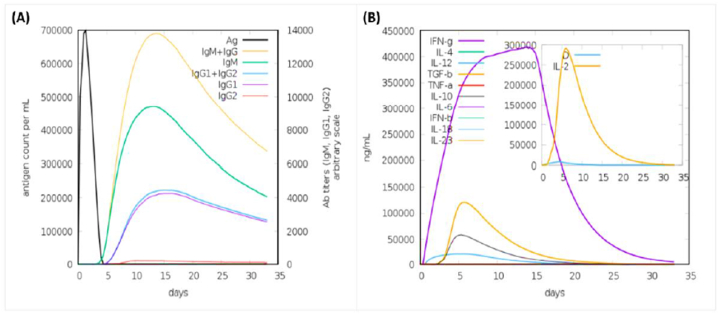
**(A)** The immunoglobulins and immunocomplexes provoked by bacteria. **(B)** The concentration of cytokines and interleukins. The Inset plot depicts the danger signal and leukocyte growth factor IL-2.

**Fig. 7:**
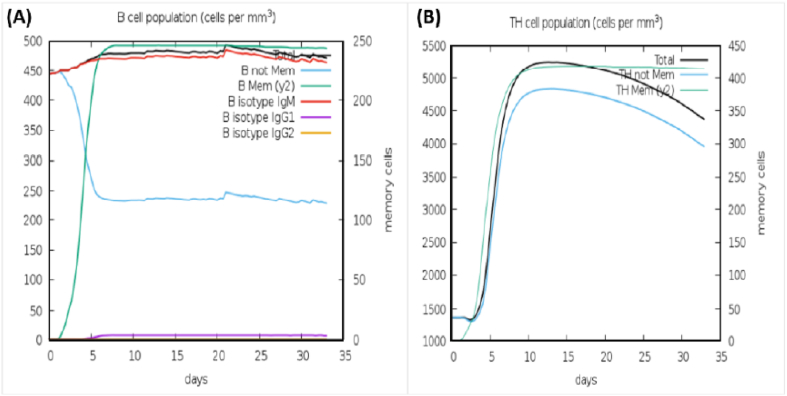
**(A)** Illustrating B Cells response **(B)** Illustrating TH Cells response.

The primary and secondary responses contribute significantly to the efficacy of the vaccine (Fig. 6A & B). shows the concentration of produced IgM and IgG antibodies in the primary response accompanied by the production of IgM, IgG1 + IgG2, and IgM + IgG antibodies in the secondary response. A graphical illustration of humoral response and high cellular population of both B cells and TH cells is shown in (Fig. 7A & B).

### 3.14 Optimization of codons and in-silico cloning

**Fig. 8:**
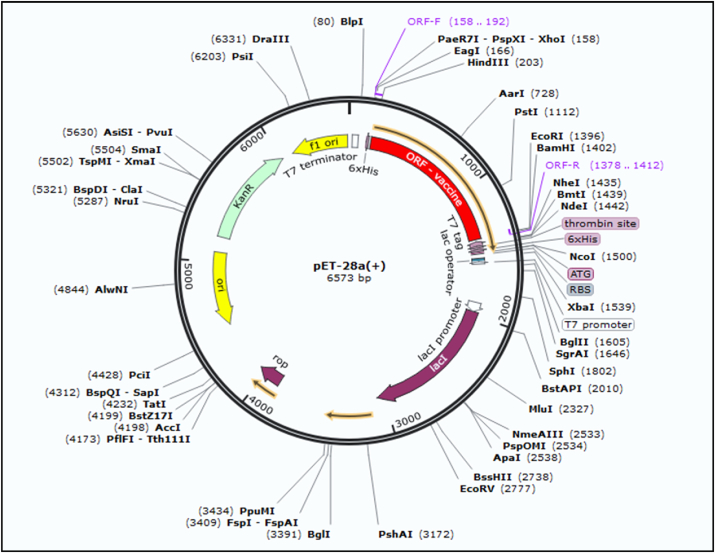
*In silico* cloning of reverse translated sequence of the vaccine into the pET28a (+) plasmid; the red portion represents the vaccine insert while the black circle is a depiction of the plasmid. This section may be divided by subheadings. It should provide a concise and precise description of the experimental results, their interpretation, as well as the experimental conclusions that can be drawn. (For interpretation of the references to color in this figure legend, the reader is referred to the Web version of this article.)

An online tool Jcat resulted in a reversely translated and optimized codon to boost up the expression of a nucleotide sequence in *E. coli* strain K12. The obtained length of the reverse translated sequence was 1245 nucleotides. The CAI score for the improved sequence was 0.95, whereas 55% GC content resulted in indicated elevated expression of the vaccine sequence in *E. coli.* Xho1 at N-terminus and EcoR1 at C-terminus were manually added into the vaccine sequence at restriction sites. SnapGene Software was employed to insert the final sequence into the pET-28a (+) plasmid to serve its cloning as shown in (Fig. 8).

## 4 Discussion

The discovery and advancements in DNA and RNA sequencing have stretched out many novel methods to expedite medical research and discoveries [50]. Antibiotic resistance to many diseases has become a serious and alarming issue of health now globally. Tuberculosis showed multiple drug resistance to 480,000 cases in 2013. This worldwide concern is predicted to cause 10 million deaths by 2050 and a loss of 100 trillion dollars [51]. However, vaccination and organ transplantation (in particular cases) is the most efficacious ways to cope with and eliminate diseases. Vaccine provokes the immune system to a current threat as well as the future; thus, is the most virtual method of obstructing infectious disease [52]. The end of Polio-Virus in Pakistan, Nigeria, and, Afghanistan has been possible only due to campaigns for Polio Vaccination [53]. Traditional vaccines confront various barriers [54]. However, computational approaches to develop vaccines minimize the time, efficacy, and cost at large [28]. It has attracted the utter attention of many scientists for defined reasons [55]. Up till now, no approved vaccine has been developed against *S. gordonii*. Synthetic vaccines have several primacies over traditional vaccines [56]. Therefore, an efficacious vaccine is required to control Infective Endocarditis convincingly. Vaccine synthesized through a computational approach lets the immune system of the host consider antigenic epitopes, leading to the refrainment of autoimmune responses and immunological reactions [56]. In fact, synthetic vaccines are more reliable than conventional [56]. This approach has been used against various pathogens such as *Klebsiella pneumonia*, *Acinetobacter baumannii*, SARS-CoV-2, microalgae, and *Staphylococcus aureus* [[57], [58], [59], [60]]. In this scientific approach, based on the scientific evidence from the literature, we selected four candidate proteins that are reportedly involved in *S. gordonii* Infection. These proteins were found to be antigenic and have a pivotal role in the bacterium's entry to the target. In the first step of this research, we subjected prioritized proteins to MHC-I and MHC-II epitopes. MHC-I and MHC-II are normally recognized by T-cells of the immune system. The binding portion of MHC in fact is identified by receptor T-cells. MHC-I molecules are found on all nucleated cells of the body and present peptides to CTL utilizing cytosolic pathway, while MHC-II presents peptides to HTL employing endocytic pathway. We constructed a vaccine sequence from multi epitopes of prioritized proteins. An adequate ratio of B cells and T cells epitopes is available in our vaccine verified by an online server for its efficacy and reliability [54]. Suitable linkers were used to join these epitopes while for enhanced immunogenicity an adjuvant was supplied. The KK linker (Lys-Lys) adds flexibility to the vaccine construct and maintains the structural integrity while granting conformation flexibility. It has wider applications and has been shown to have increased the stability and antigenicity of the virus-like particles (VLPs), subunit and DNA vaccines. The AAY (Ala-Ala-Tyr) linker on the other hand, having the hydrophobic nature increases the structural stability and also induces a stronger Th1 immune response which is a pre-requisite for the clearance of various pathogens. The GPGPG (Gly-Pro-Gly-Pro-Gly) linker though exhibits a rigid surface and is primarily used for domain separation in a MEVC. It also improves the accessibility of the epitopes to the immune system [61,62]. The reasons for the usage of mammalian adjuvant include the enhanced antigen presentation shown by the dendritic cells which consequently leads to the activation of CTL response and a stronger immune reaction to the antigen. Additionally, it robustly activates the toll-like receptors (TLRs) which as a result produce cytokines and chemokines that cause immune cell activation and recruitment. Moreover, it induces the Th1 immune response which is essential for the clearance of intracellular pathogens. Furthermore, this adjuvant is safe and well-tolerated as it is a part of the natural system of the human body that possesses safe morphology [[63], [64], [65]]. The molecular weight of the vaccine is 42 KDa, which supports the perfect range for a vaccine. The acceptable range is between 30 KDa and 60 KDa. Our vaccine has an alkaline nature with a score of theoretical PI 10. The vaccine scored 26.31 for the aliphatic index, which shows its thermal stability; the required range is between 0 and 40. The Secondary and Tertiary structure of a vaccine illustrates the plain function of a protein. These structures were constructed by online servers PSIPRED V3.3, SOPMA, and Robetta, respectively. The efficacy of the tertiary structure was confirmed using the ProCheck server. Ramachandran plot of a vaccine construct revealed most residues in the favored region to finalize the research. The ClusPro server was exploited that docked the vaccine with Human TLR2 (Toll-Like Receptor). The result revealed 10 docking complexes, presenting the number of hydrogen bonds, salt bridges, and other essential interactions between residues of vaccine and human TLR2. Among 10, the-best docked model was selected and finalized for further evaluation. Hence, Jcat tool served the reverse translation of peptide sequence and codon optimization. Whereas SnapGene software was opted to insert the resultant nucleotide sequence into pET28 (+) plasmid for cloning. The choice of vector is based on the availability of T7 a stronger promoter for the expression in *E. coli* system. Moreover, it also has the advantage of high-level of expression of the inserted gene. Due to the availability of N-terminal polyhistidine (6xHis) tag this promotor allows easy downstream processing and contain multiple unique restriction sites. It also owns the advantage of carrying antibiotics resistance gene which help in the selection of chimeric bacteria. Furthermore, it is a versatile choice as it can be used for both prokaryotic and eukaryotic group of organisms. pET28a has been extensively used and characterized, which means that there is a lot of information available on its performance, optimization, and troubleshooting. This makes it a reliable choice for protein expression experiments [66,67]. On the other hand, the pET30a with the limited selection of fusion tags and few restriction sites makes it unsuitable for the cloning of large fragment. Moreover, the availability of pET30a is also limited and due to single antibiotics resistance gene the selection of different bacterial strains resistant to different genes may need other markers [68]. Hence, pET28a is was the best choice to clone our vaccine construct. The experimental validation stands the only limitation of the current study. For this purpose, the Peripheral blood mononuclear cells (PMBC) can be used for immune cell phenotyping and antibody testing. Moreover, in presence of soluble anti-CD3 antibody PMBC will be cultured to determine the T cell proliferation while the cytokines producing T cells can be investigated through Intracellular cytokine staining (ICS). Finally, the Western blot and co-immunoprecipitation methods can be used for interactions determination with the HLA molecules. The developed vaccine sequence manifests supplementary wet lab validation to coup the emerging infection caused *S. gordonii.*

## 5 Conclusions

In this scientific study, a computational approach, specifically an immune-informatics strategy was adopted to construct and design a reliable multi-epitope subunit vaccine that can be efficacious against Infective Endocarditis. The first step addressed was the retrieval of four in-vivo reported proteins GspB, uniport ID (Q939N5), CshA (A8AWJ3), CshB (A8AXC5), andSspA (A8AUS0), accompanied by the selection of CTL, HTL and B-cell epitopes; epitopes were fastened by appropriate linkers. The reliability of the vaccine was maintained by analyzing antigenic, allergenic, toxic, and physiochemical properties. Molecular docking between Human TLR2 and the vaccine was carried out to select the best stable docked complex with maximum hydrogen bonds and salt bridges. The peptide sequence was reversely translated, optimized, and inserted into plasmid pET28a + for cloning to validate its expression and stability. The immune simulation confirmed the immune response triggered by the constructed multi-epitope subunit vaccine. Consequently, this study warrants further practical validation in a wet lab which stands as the only limitation of the current study. It will incite a long-standing immunity and will aid in controlling *S. gordonii-associated* infections.

## Funding information

This research is supported by grants from the 10.13039/501100001809National Science Foundation of China (Grant No.32070662, 61832019, 32030063)， the Key Research Area Grant 2016YFA0501703 of the Ministry of Science and Technology of China, the 10.13039/501100003399Science and Technology Commission of Shanghai Municipality (Grant No.: 19430750600), as well as 10.13039/501100004921SJTU JiRLMDS Joint Research Fund and Joint Research Funds for Medical and Engineering and Scientific Research at 10.13039/501100008233Shanghai Jiao Tong University (YG2021ZD02).

## Data availability statement

Data will be made available on request.

## Additional information

No additional information is available for this paper.

## Author contributions

**Conceptualization:** Syed Nouman Nasir, Ayesha Iftikhar, Farukh Zubair; **Data curation:** Syed Nouman Nasir, Ayesha Iftikhar, Farukh Zubair, Abdulrahman Alshammari, Metab Alharbi, Yasir Waheed, Abbas Khan and Liaqat Ali; **Conceived and designed the experiments:** Syed Nouman Nasir, Abbas Khan, Syed Shujait Ali, Muhammad Waseem, Liaqat Ali and Abbas Khan; Funding **acquisition:** Abdulrahman Alshammari, Metab Alharbi; **Investigation:** Abdulrahman Alshammari, Metab Alharbi, Abdullah F. Alasmari; **Performed the experiments:** Syed Nouman Nasir, Abbas Khan, Ayesha Iftikhar, Farukh Zubair; Abdulrahman Alshammari, Abdullah F. Alasmari, Metab Alharbi; **Analyzed and interpreted the data:** Abbas Khan and Liaqat Ali; Dong-Qing Wei, Yasir Waheed; Software, Syed Shujait Ali, Abdulrahman Alshammari, and Syed Shujait Ali; **Supervision:** Dong-Qing Wei and Syed Shujait Ali; **Contributed reagents, materials, analysis tools or data:** Metab Alharbi, Abbas Khan, Dong-Qing Wei and Syed Ali; Abdullah F. Alasmari and Abbas Khan; **Wrote the paper:** Syed Nouman Nasir, Dong-Qing Wei and Abbas Khan; **Writing – review & editing**: Syed Shujait Ali and Dong-Qing Wei.

## Declaration of competing interest

Authors declare there is no declaration of interest.
